# Lactate Signal: Modulator of Cellular Energy Production and Anabolism

**DOI:** 10.1002/advs.78115

**Published:** 2026-10-08

**Authors:** Han Wang, Si‐Yuan Yang, Wei Xu

**Affiliations:** ^1^ The Obstetrics & Gynecology Hospital of Fudan University Shanghai Key Lab of Reproduction and Development Shanghai Key Lab of Female Reproductive Endocrine Related Diseases Shanghai Key Laboratory of Metabolic Remodeling and Health Institute of Metabolism and Integrative Biology and State Key Laboratory of Genetics and Development of Complex Phenotypes Fudan University Shanghai People's Republic of China

**Keywords:** anabolism, catabolism, cell biology, glycolysis, lactate/pyruvate, metabolic pathway, signal transduction

## Abstract

Lactate is no longer viewed simply as a glycolytic end‐product, but as a compartmentalized signaling metabolite that coordinates energy production, carbon redistribution, redox balance, and anabolic commitment. This review discusses lactate as a regulator of the catabolism‐anabolism axis, emphasizing two major patterns of molecular interpretation. First, lactate acts through non‐covalent mechanisms, including transporter‐mediated flux, receptor‐dependent sensing, pH‐linked effects, and direct binding to intracellular proteins. These processes allow lactate‐rich states to rapidly couple metabolic flux to signaling pathways. Second, lactate‐associated metabolic states are translated into the dynamic and reversible change of covalent post‐translational modifications, including histone and non‐histone lactylation. Histone lactylation connects glycolytic metabolism to transcriptional regulation, whereas non‐histone lactylation expands lactate‐dependent control to immune signaling, mitochondrial metabolism, DNA repair, cardiovascular stress, tissue remodeling, and cancer progression. We further discuss how extracellular, cytosolic, mitochondrial‐associated, and nuclear lactate pools provide distinct biochemical contexts in which lactate‐dependent mechanisms can operate. By integrating lactate transport, receptor sensing, protein binding, metabolism, and lactylation, this Review uses compartmental organization as a framework for synthesizing how lactate‐rich states influence metabolic adaptation, stress responses, immune regulation, tissue remodeling, disease progression, and, in selected contexts, anabolic or reparative programs.

## Graphical abstract

Compartmentalized lactate signaling provides a spatial framework for understanding how lactate‐rich states are generated, transported, sensed, and translated into biological outputs. This Review integrates extracellular, cytosolic, mitochondrial‐associated, and nuclear lactate mechanisms to explain how lactate shapes metabolic adaptation, immune regulation, tissue remodeling, disease progression, and therapeutic response.

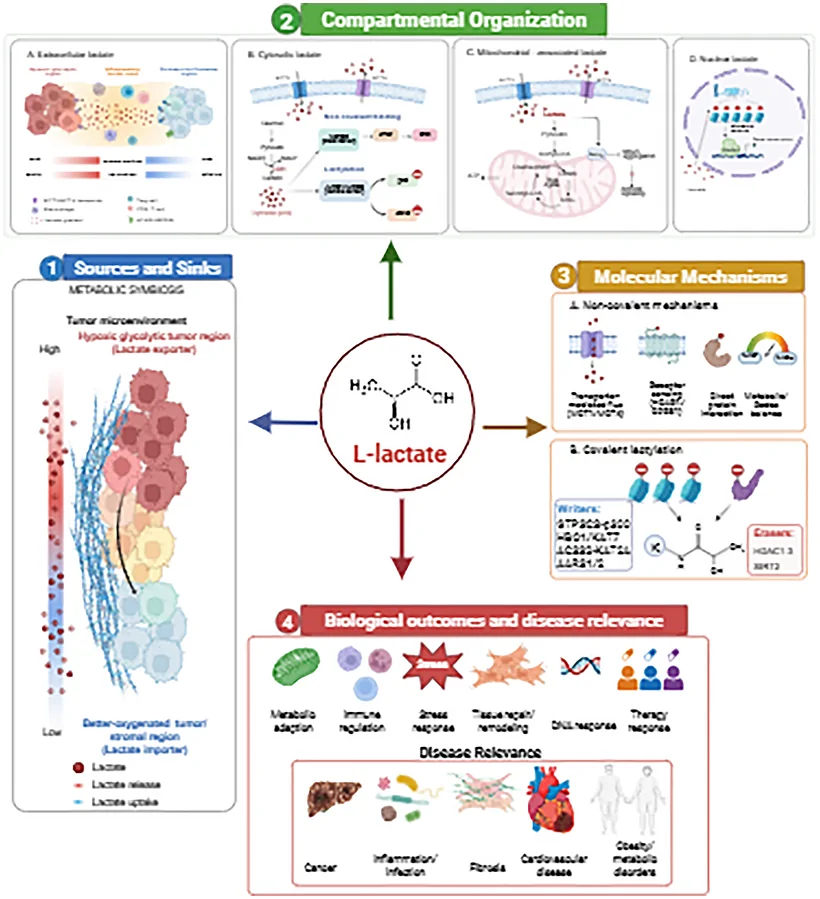

## 1 Introduction

Historically, lactate was often described as a terminal product of anaerobic glycolysis associated with oxygen limitation and metabolic inefficiency [1]. Lactate dehydrogenase (LDH) catalyzes the reversible reduction of pyruvate to lactate with oxidation of NADH to NAD^+^, and the net lactate production occurs under fully aerobic as well as hypoxic conditions whenever glycolytic pyruvate and cytosolic reducing equivalents exceed their immediate oxidative handling or the cellular redox state favors the LDH reaction [2, 3, 4]. The lactate shuttle framework encompasses organ–organ, cell‐cell, and intracellular lactate shuttling, linking glycolytic and oxidative metabolism across tissues, cells, and intracellular compartments [2, 3, 5]. Chemically, the product of the LDH reaction is lactate, not undissociated lactic acid; because the pKa of lactic acid is approximately 3.86, more than 99% is present as lactate at physiological pH. Accordingly, this Review uses ‘lactate’ for the physiological species and reserves ‘lactic acid’ for explicitly acidified experimental preparations or when describing the terminology used in a cited study. Unless otherwise specified, lactate refers to the predominant mammalian enantiomer, *L*‐lactate.

A key feature of lactate organization is its compartmental distribution. Local lactate abundance is shaped by the balance between production, export, uptake, oxidation, perfusion, pH buffering, and transporter expression [6, 7, 8, 9, 10]. Glycolytically active cells can release lactate under both fully aerobic and hypoxic conditions, whereas oxidative cells can import and utilize it as fuel [2, 3, 6, 8, 10, 11]. Consequently, lactate gradients emerge across extracellular spaces, between neighboring cell populations, and potentially among intracellular compartments. Such gradients are particularly evident in metabolically active or stressed tissues, including exercising muscle, brain, inflamed tissues, and tumors [3, 10, 12, 13, 14, 15]. Thus, the biological function of lactate depends on where it is generated, where it accumulates, and what cells or organelles are exposed to it [6, 7, 10, 16].

This spatial organization places lactate at the interface between ATP‐generating catabolism and ATP‐consuming anabolism. During high glycolytic flux, lactate production supports ATP generation by regenerating NAD^+^ and sustaining glycolysis [4, 15]. At the same time, lactate accumulation and redistribution can signal that a cell or tissue has entered a high‐flux metabolic state [17, 18]. Depending on cellular context, lactate‐rich states may influence metabolic adaptation, immune regulation, stress responses, transcriptional remodeling, tissue repair, and anabolic programs such as proliferation or biosynthesis [11, 17, 18, 19]. In this sense, lactate is not only a byproduct of catabolic metabolism but also a compartmental cue capable of influencing the balance of anabolic, reparative, and adaptive programs.

For lactate to exert such regulatory effects, cells must possess mechanisms to read lactate‐rich states. Current evidence supports two major molecular modes of lactate interpretation. The first is non‐covalent lactate sensing and binding, including transporter‐dependent lactate uptake, receptor‐mediated extracellular sensing, and direct regulation of lactate‐responsive proteins such as NDRG3 and MAVS [8, 17, 20, 21, 22]. These mechanisms allow rapid and reversible responses to local lactate concentration, pH, and metabolic context [11, 14, 21, 23, 24]. The second is covalent lactate‐associated protein modification, prominently lysine lactylation, which in mammalian systems largely refers to *L‐*lactate‐derived *L‐*lactylation [18, 19, 25, 26, 27]. Histone lactylation links lactate accumulation to chromatin remodeling and transcriptional regulation, whereas non‐histone lactylation may extend lactate‐dependent regulation to broader proteome function [16, 25, 27, 28, 29, 30].

In this Review, we use compartmental organization as an integrative framework to organize current evidence on lactate biology. This framework is not intended to define a new signaling paradigm or imply that compartmental lactate signaling is already established as a consensus principle. Rather, it provides a way to connect independently reported mechanisms, including MCT‐mediated transport, HCAR1/GPR81‐dependent sensing, direct regulation of lactate‐responsive proteins, and lactylation‐dependent protein regulation. We propose that this compartment‐oriented framework helps explain how lactate‐rich states can connect metabolic flux with stress adaptation, immune regulation, tissue remodeling, and, in selected contexts, anabolic or reparative programs. We first discuss the lactate landscape, including sources, sinks, transporters, and gradients; then summarize non‐covalent lactate sensing/binding and lactylation; and finally organize lactate signaling across extracellular, cytosolic, mitochondrial‐associated, and nuclear compartments in physiology and disease.

## 2 The Lactate Landscape: Sources, Sinks, and Transport

### 2.1 Lactate Sources and Sinks Across Tissues

Lactate is generated primarily through the reversible reduction of pyruvate by LDH, a reaction coupled to the oxidation of NADH to NAD^+^ [2, 4]. The reaction supports cytosolic redox balance and sustains glycolysis when glycolytic flux is high, but it is not a specific marker of oxygen deficiency. Lactate is produced continuously under aerobic conditions [2, 3, 4], and hypoxia can amplify lactate accumulation by constraining oxidative metabolism and inducing glycolytic and transporter programs, but oxygen limitation is neither required for LDH activity nor sufficient to define lactate physiology [2, 3, 5, 8]. Thus, lactate production can coexist with robust mitochondrial respiration and can reflect redox balancing, rapid ATP demand, or biosynthetic metabolism rather than mitochondrial failure [2, 4, 6, 17, 18, 20].

Lactate‐consuming compartments are equally important. Oxidative tissues such as heart, skeletal muscle, brain, liver, and kidney can take up lactate and convert it to pyruvate for mitochondrial oxidation or gluconeogenesis [2, 3, 7]. These producer‐consumer relationships are central to the lactate shuttle; that is, lactate generated in one cell, tissue region, or organ becomes an oxidative or gluconeogenic substrate in another, and intracellular shuttling links cytosolic production to mitochondrial disposal [2, 3, 5]. The mitochondrial reticulum contributes to this organization by providing a distributed respiratory sink that helps maintain concentration gradients from lactate‐producing regions toward oxidative sites, and this concept is supported by isotope‐tracer studies, tissue exchange measurements, and mitochondrial‐reticulum preparations [2, 5].

This source‐sink organization is especially relevant in pathophysiological tissues. In tumors, hypoxic and highly glycolytic regions often release lactate, whereas better oxygenated tumor cells or stromal cells may consume lactate as an oxidative fuel [6, 8, 10, 11]. Glycolytic tumor cells, represented by WiDr colorectal adenocarcinoma cells, function as lactate‐producing compartments by relying on glucose‐driven glycolysis and releasing lactate into the extracellular environment. In contrast, oxidative tumor cells, represented by SiHa cervical squamous carcinoma cells, act as lactate‐consuming compartments, importing extracellular lactate through MCT1 and utilizing it as an oxidative substrate through mitochondrial metabolism. Thus, oxygen availability and intrinsic metabolic phenotype determine whether lactate functions primarily as an exported metabolic product or an imported fuel source. This lactate exchange establishes a metabolic symbiosis between hypoxic/glycolytic and oxygenated/oxidative tumor cell populations, in which lactate produced by glycolytic cells supports oxidative metabolism and ATP generation in neighboring oxidative cells [10]. Human non‐small‐cell lung tumors also use lactate as a TCA‐cycle carbon source in vivo, supporting the physiological relevance of tumor lactate consumption [6]. Thus, lactate gradients are not passive byproducts; they represent spatial biochemical contexts generated by metabolic heterogeneity and transporter‐dependent exchange, which can influence tissue communication, nutrient competition, and signaling states (Figure 1A).

**FIGURE 1:**
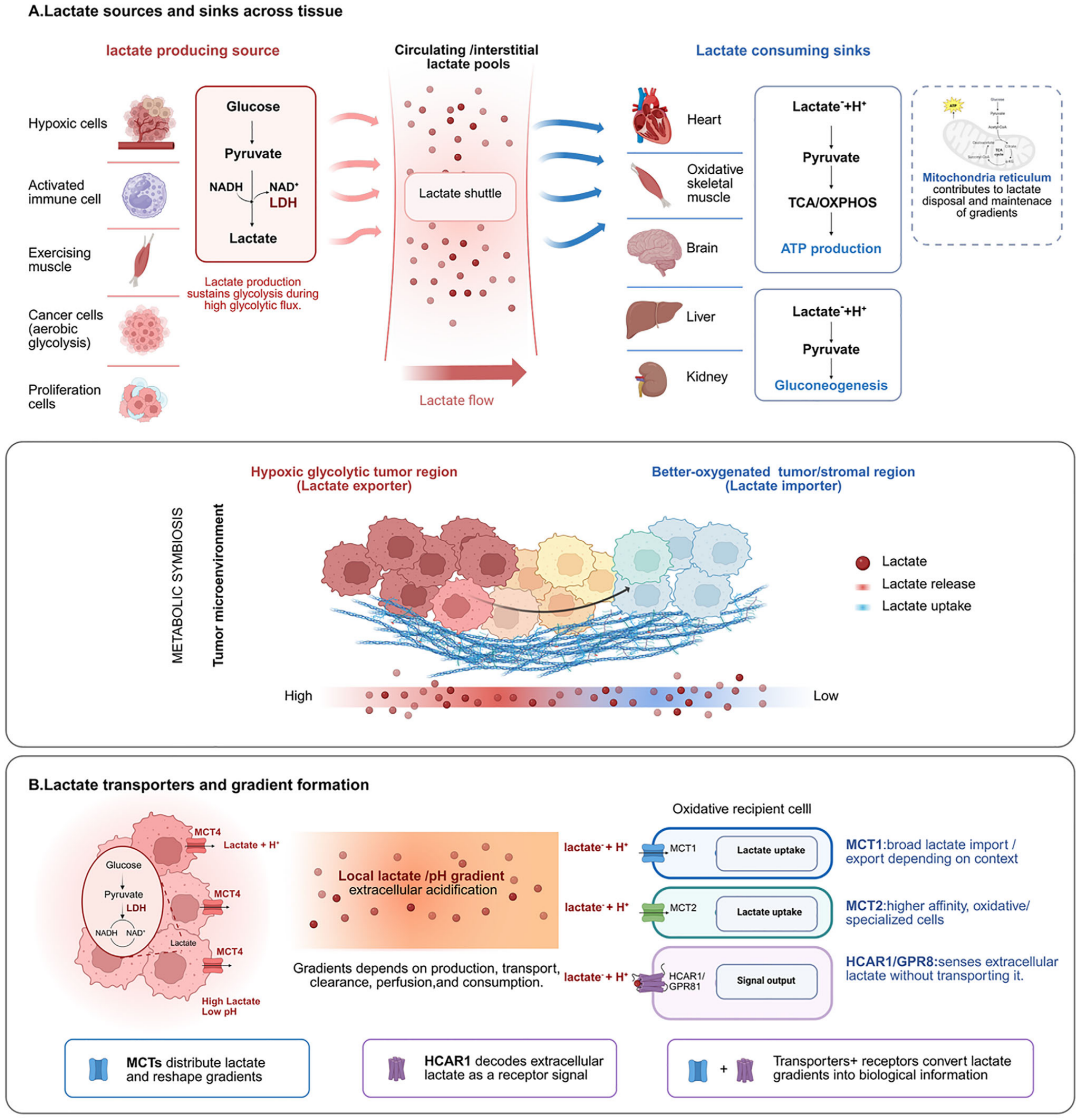
The lactate landscape: sources, sinks, transporters, and receptor‐mediated sensing. (A) Lactate sources and sinks across tissues. LDH produces lactate from pyruvate with NADH oxidation and NAD^+^ regeneration under both aerobic and hypoxic conditions. Released lactate forms circulating and interstitial pools that can be utilized by recipient tissues, including heart, oxidative skeletal muscle, brain, and oxidative tumor or stromal cells, where lactate‐derived carbon enters mitochondrial oxidation after conversion to pyruvate. Lactate also serves as a gluconeogenic precursor in the liver and kidney. In tumors, metabolically heterogeneous regions establish producer‐consumer relationships that support metabolic cooperation among tumor, stromal, and immune cells. Panel A synthesizes the Lactate Shuttle framework [2, 3, 5] with tissue‐ and tumor‐tracing evidence [6, 7, 10]. (B) Lactate transporters and gradient formation. Proton‐coupled MCTs/SLC16 transporters mediate bidirectional lactate/proton flux, whereas sodium‐coupled SLC5A8 and SLC5A12 provide distinct uptake routes in selected epithelia and immune cells. Extracellular lactate can also be sensed by HCAR1/GPR81 without transport into the cytosol. Together, production, clearance, perfusion, consumption, transporter activity, and receptor sensing establish and interpret spatial lactate gradients. Figure created using BioRender.com.

### 2.2 Lactate Transporters and Gradient Formation

Because lactate exists largely as a charged monocarboxylate at physiological pH, its movement across membranes depends heavily on transporters [31, 32]. The major lactate transporters are members of the solute carrier 16 family, particularly MCT1/SLC16A1, MCT2/SLC16A7, MCT3/SLC16A8, and MCT4/SLC16A3 [31, 32]. These transporters mediate proton‐coupled monocarboxylate transport, meaning that lactate movement is linked to proton flux and pH regulation [32, 33].

MCT isoforms have distinct but overlapping physiological roles. MCT1 is broadly expressed and can mediate lactate uptake or export depending on the direction of lactate and proton gradients [9, 10, 32]. MCT2 is a high‐affinity lactate transporter enriched in metabolically specialized tissues such as the nervous system, where MCT2 expression has been linked to astrocyte‐neuron metabolic coupling and brain function [12, 13, 34]. MCT4 is frequently associated with highly glycolytic cells and supports lactate export under conditions of elevated glycolytic flux or hypoxia [8, 9]. In tumors, MCT1 and MCT4 can cooperate to sustain lactate shuttling, extracellular acidification, and metabolic adaptation [9, 10, 35, 36].

The direction of lactate transport is not determined by transporter identity alone. It depends on intracellular and extracellular lactate concentration, proton concentration, metabolic consumption, transporter abundance, and tissue perfusion. Therefore, lactate gradients represent integrated metabolic and physicochemical gradients [10, 11]. In lactate‐producing cells, export of lactate and protons can help maintain intracellular pH while acidifying the extracellular environment [8, 9]. In lactate‐consuming cells, import of lactate can provide oxidative substrate and metabolic information. This transporter‐dependent spatial redistribution is a prerequisite for lactate signaling because it determines which cells and compartments are exposed to lactate and for how long [7, 9, 10].

In addition to transporter‐mediated lactate movement, extracellular lactate gradients can also be interpreted through lactate‐sensing receptors [21, 22, 37]. The best‐characterized lactate receptor is hydroxycarboxylic acid receptor 1, also known as HCAR1 or GPR81 [21, 22]. Unlike MCTs, which directly mediate lactate flux across membranes, HCAR1 functions as a plasma membrane G protein‐coupled receptor that allows cells to sense extracellular lactate without necessarily transporting it into the cytosol [21, 22, 37]. Thus, MCTs mainly determine where lactate moves, whereas HCAR1 helps determine how cells interpret extracellular lactate‐rich environments [10, 21, 37]. Proton‐coupled SLC16 transporters distribute lactate and shape lactate/pH gradients, whereas HCAR1 decodes extracellular lactate as a receptor signal [1, 2, 6, 7, 8, 12, 13, 18, 20, 22, 37]. A second transporter system comprises the sodium‐coupled monocarboxylate transporters SMCT1/SLC5A8 and SMCT2/SLC5A12, which use the Na^+^ electrochemical gradient rather than proton cotransport. SLC5A8 is a high‐affinity transporter expressed prominently in the colon and kidney, where it participates in epithelial uptake of lactate and other monocarboxylates and links microbial or host metabolites to mucosal and metabolic homeostasis [38, 39]. SLC5A12 is a lower‐affinity transporter expressed in kidney, intestine, and selected immune cells. In activated CD4^+^ T cells, it mediates sodium‐lactate uptake, promotes retention within inflamed tissues, and supports pro‐inflammatory metabolic reprogramming [40]. Recent work further shows that SLC5A12‐dependent lactate signaling promotes ectopic lymphoid aggregation, whereas SLC5A12 blockade reduces inflammatory cytokines, enhances T cell, and disrupts T/B‐cell segregation in Sjögren disease [40]. Thus, proton‐ and sodium‐coupled transporters are physiologically distinct routes by which lactate flux influences carbon use, ion gradients, pH, immune‐cell localization, and tissue organization.

## 3 Lactate Signal: Non‐Covalent Interactions and Lactylation

Lactate signaling can be organized into two major molecular patterns: non‐covalent lactate interactions and covalent lactate‐associated post‐translational modification (PTM). The previous section establishes how lactate gradients are generated and distributed through source‐sink relationships and transporter‐dependent exchange. Here, the emphasis shifts from landscape to mechanisms by which cells interpret lactate‐rich states once lactate is present in their vicinity or interior. Non‐covalent mechanisms encompass transporter‐mediated lactate access, receptor sensing, and direct regulation of lactate‐responsive proteins, alongside secondary influences such as redox state and pH fluctuations. These responses are typically rapid, reversible, and concentration‐dependent, and are critically shaped by local lactate gradients, transporter abundance, and cellular context. In contrast, covalent lactylation can dynamically translate lactate‐associated metabolic states into histone and non‐histone protein modifications, producing long‐lasting effects on transcription, protein function, and cellular identity. This section focuses on the molecular players and biochemical processes themselves, while their compartmentalized outcomes will be discussed in Section 4.

### 3.1 Non‐Covalent Lactate Interactions

The first layer of non‐covalent lactate signaling is receptor‐based sensing. Extracellular lactate can be decoded by receptor‐based sensing. HCAR1/GPR81 is a classical lactate‐sensing G protein‐coupled receptor that detects extracellular lactate without transporting it, as discussed above. In adipocytes, lactate activates GPR81 and suppresses lipolysis [21], induces chemokine expression in tumor cells [37], and modulates neuronal network activity [41], establishing extracellular lactate as an endogenous receptor ligand that can restrain catabolic substrate mobilization [21, 22].

Beyond transporters and receptors, lactate can directly regulate intracellular proteins through non‐covalent mechanisms. Under prolonged hypoxia, lactate stabilizes NDRG3 by preventing PHD2/VHL‐dependent degradation; stabilized NDRG3 interacts with c‐Raf and activates Raf‐ERK signaling, promoting angiogenesis and cell growth in Huh‐1 cells [17]. Lactate regulates cell‐cycle progression by remodeling the anaphase‐promoting complex/cyclosome (APC/C), thereby controlling mitotic progression [42]. Lactate also directly binds the transmembrane domain of MAVS, prevents MAVS aggregation, suppresses RIG‐I‐like receptor signaling, and reduces type I interferon production [20]. Additionally, transporter‐mediated lactate flux can create local pH changes and redox shifts that influence protein conformation and signaling complex assembly primarily via distribution mechanisms [14, 15]. Thus, non‐covalent lactate recognition can directly couple lactate‐rich states to rapid signaling outputs and, in specific enzyme systems, to downstream covalent lactylation.

### 3.2 Covalent Lactylation

The second major molecular mode of lactate signaling is covalent lactylation. Covalent protein modification by lactate was first reported in a pan‐metabolite screening [43, 44], and histone lysine lactylation was first identified as a lactate‐derived epigenetic modification that directly stimulates gene transcription from chromatin [18, 45]; hypoxia and bacterial challenge increase glycolytic lactate production and induce histone lactylation, and late‐phase macrophage histone lactylation promotes homeostatic and wound‐healing genes such as Arg1 [18]. Subsequent primary studies extended histone lactylation into tumor immunity, fibrosis, development, regeneration, trained immunity, and tissue protection. For example, lactylation‐driven METTL3‐mediated m6A modification promotes immunosuppression of tumor‐infiltrating myeloid cells; HK2‐mediated histone lactylation drives hepatic stellate‐cell activation and liver fibrosis; glycolysis‐regulated histone lactylation couples embryonic metabolic state to chromatin organization; and macrophage histone lactylation predicts gene‐expression changes during ischemia‐induced muscle regeneration [29, 30, 46]. Lactate also contributes to trained immunity through TCA‐cycle fueling and histone lactylation, while exercise‐induced histone lactylation in monocyte‐derived macrophages protects against sepsis‐induced cardiomyopathy [47]. These studies support the idea that histone lactylation converts lactate‐rich metabolic states into transcriptional programs involved in DNA repair, chromatin remodeling, trained immunity, fibrosis, and disease.

Non‐histone lactylation expands lactate signaling from chromatin to the broader proteome. At the level of metabolic regulation, it regulates protein activity, stability, localization, and interaction networks. For instance, lactylation of the glycolytic enzyme PKM2 suppresses inflammatory metabolic adaptation [48], while lactylation of DNA repair proteins MRE11 and NBS1 promotes homologous recombination repair and therapy resistance [49, 50]. Mitochondrial protein lactylation, such as that of PDHA1 and CPT2 under hypoxia, can restrain oxidative phosphorylation under hypoxic conditions [16]. In colorectal cancer and therapy resistance, eEF1A2 K408 lactylation enhances translation elongation and protein synthesis [51], RPA1 K88 lactylation promotes DNA repair and radioresistance, and DCBLD1 lactylation stabilizes DCBLD1 to activate the pentose phosphate pathway through G6PD [52]. In cellular stress responses and structural integrity, lactylation has been implicated in cardiovascular protection. α‐myosin heavy chain K1897 lactylation maintains sarcomeric structure and alleviates heart failure, whereas Serpina3k/SERPINA3 K351 lactylation protects against cardiac ischemia‐reperfusion injury [53]. These examples demonstrate that lactylation is a versatile PTM capable of tuning diverse protein functions across cellular compartments, the consequences of which are explored in Section 4.

Lactylation is dynamically regulated by writer and eraser systems. The original histone lactylation study implicated p300 as an early candidate writer [18]. More recent work has expanded the writer landscape. HBO1/KAT7 catalyzes lysine lactylation and mediates H3K9la‐dependent transcription [54]; ACSS2 acts as a lactyl‐CoA synthetase and couples with KAT2A to drive histone lactylation and tumor immune evasion [55]. In glioblastoma cells, AARS1 functions as a lactate sensor and lactyltransferase that modifies p53 [25], and AARS1/AARS2 act as lactate‐sensing lactyltransferases that regulate cGAS [27, 56]. In gastric cancer, lactate promotes AARS1 nuclear translocation and tumor‐cell proliferation via YAP K90 and TEAD1 K108 lactylation [19]. HDAC1‐3 have been identified as histone lysine deacetylases [26]. SIRT2 functions as a histone delactylase that suppresses neuroblastoma‐cell proliferation and migration [56], SIRT3 reverses selected mitochondrial lactylation marks, and SIRT6 has also been described as a histone delactylase [57]. These writer and eraser studies indicate that lactylation is not merely a passive consequence of lactate accumulation, but a regulated lysine acylation system controlled by lactate availability, lactyl‐donor generation, enzyme localization, substrate accessibility, and metabolic state. Together, non‐covalent lactate interactions and covalent lactylation form a layered molecular interpretation system. MCTs distribute lactate and establish metabolic access, HCAR1/GPR81 senses extracellular lactate as a receptor signal, intracellular proteins such as NDRG3 and MAVS respond directly to lactate in selected contexts, and histone or non‐histone lactylation can link lactate‐associated metabolic states to transcriptional or proteomic regulation.

Although lactylation has emerged as an important mechanism linking lactate‐associated metabolism to chromatin regulation and protein function, several key issues remain unresolved. First, the physiological lactyl donor is still not fully defined. Histone lactylation was originally linked to lactate accumulation and p300 activity [18], whereas more recent studies suggest additional donor‐generating or writer‐associated pathways, including ACSS2‐KAT2A, nuclear GTPSCS‐p300, and AARS‐family enzymes [58]. These mechanisms may operate in different compartments, cell types, or metabolic states rather than representing a single universal pathway. Second, the specificity of lactylation writers and erasers requires further clarification. Candidate writers and writer‐associated systems include p300, GTPSCS‐p300, HBO1/KAT7, ACSS2‐KAT2A, AARS1, AARS2, HAT1, and KAT8, whereas HDAC1‐3, SIRT2, SIRT3, and SIRT6 have been reported to remove selected lactylation marks [45, 55, 56, 57, 58, 59, 60]. However, it remains important to distinguish enzymes that directly catalyze lactyl transfer from those that indirectly influence lactylation by altering lactate abundance, lactyl‐donor availability, chromatin accessibility, or protein complex formation. Third, many reported lactylation events still require quantitative and functional validation. Enrichment‐based mass spectrometry and pan‐lactyl‐lysine antibodies are useful for discovery, but they do not always establish site occupancy, stoichiometry, stereochemistry, or functional dominance over other lysine acylations such as acetylation. Therefore, newly identified lactylation targets should be validated using quantitative proteomics, isotopic tracing, site‐specific mutagenesis, rescue experiments, and orthogonal biochemical approaches. This is particularly important for non‐histone lactylation events implicated in mitochondrial metabolism, DNA repair, immune regulation, and therapy resistance [16, 61, 62, 63] (Table 1).

**TABLE 1:** Representative Downstream Effects of Lactylation.

Category	Lactylated target/mark	Context	Evidence‐supported downstream effect	Functional interpretation
Histone lactylation	Global histone lysine lactylation, including multiple core‐histone Kla sites [18]	Bacterially challenged M1 macrophages; hypoxia and lactate‐rich glycolytic states	Lactate‐derived histone lactylation directly stimulates transcription from chromatin; late‐phase macrophage histone lactylation induces homeostatic and wound‐healing genes such as Arg1	Foundational evidence that lactate can be converted into a chromatin modification linking glycolytic metabolism to transcriptional repair programs
	H3K18la [30]	Tumor‐infiltrating myeloid cells	Lactylation‐driven METTL3 upregulation promotes RNA m6A modification and activates JAK1–STAT3 signaling, leading to myeloid‐cell immunosuppression	Histone lactylation links tumor lactate metabolism to epitranscriptomic remodeling and immunosuppressive myeloid states
	HK2‐associated histone lactylation [29]	Hepatic stellate‐cell activation and liver fibrosis	HK2‐dependent glycolysis increases histone lactylation and drives profibrotic gene expression required for stellate‐cell activation	Histone lactylation converts glycolytic reprogramming into fibrogenic transcriptional activation
	H3K18la at tissue‐specific active enhancers [64]	Mouse and human tissues; multi‐tissue enhancer profiling	H3K18la marks tissue‐specific active enhancers and correlates with enhancer‐associated gene regulation	Lactylation can act as a spatially patterned enhancer‐associated chromatin mark rather than only a global lactate‐stress signal
	Glycolysis‐regulated histone lactylation [65]	Embryonic development and cell‐state transitions	Histone lactylation couples embryonic metabolic state to chromatin organization and developmental gene‐regulatory networks	Lactylation links glycolytic metabolism to developmental transcriptional plasticity
	Histone lactylation in macrophages [46]	Ischemia‐induced muscle regeneration	Macrophage histone lactylation dynamics predict gene‐expression changes during tissue regeneration	Lactylation participates in metabolic control of macrophage reparative programs
	H3K18la recognized by Brg1 [66]	Early induced pluripotent stem‐cell reprogramming	Dux‐induced H3K18la recruits Brg1 and promotes early reprogramming‐associated transcriptional programs	Histone lactylation can function as a reader‐interpreted chromatin signal during cell‐fate reprogramming
	H3K14la at regulatory regions controlling NEDD4 [67]	Oxaliplatin‐ and 5‐FU‐resistant hepatocellular carcinoma	H3K14la increases NEDD4 expression; NEDD4 promotes PTEN ubiquitination and degradation, reinforcing glycolysis and multidrug resistance	Histone lactylation creates a glycolysis‐NEDD4‐PTEN positive‐feedback loop that stabilizes chemotherapy‐resistant metabolic states
	H3K18la at the RUBCNL/Pacer locus [68, 69]	Colorectal cancer under bevacizumab pressure	Tumor‐derived lactate increases H3K18la and RUBCNL expression, promoting autophagosome maturation and resistance to bevacizumab	Lactate‐rich therapy pressure is converted into an autophagy‐dependent survival program
	H4K12la [70]	Triple‐negative breast cancer	Lactate‐induced H4K12la suppresses SLFN5 promoter activity and reduces SLFN5 expression, supporting malignant progression	Histone lactylation can silence tumor‐suppressive transcriptional programs and promote aggressive cancer behavior
	H4K5la [71]	Neuroblastoma with DNAJC12 downregulation	Increased H4K5la upregulates COL1A1 transcription and enhances invasive behavior; high H4K5la correlates with poor clinical outcome	Glycolysis‐linked histone lactylation connects metabolic reprogramming to extracellular‐matrix and invasion programs
	H3K18la at the NOD2 locus [72]	Bilirubin encephalopathy model; UCB‐treated astrocytes	H3K18la increases NOD2 expression, activating MAPK/NF‐κB signaling and promoting astrocyte pyroptosis	Histone lactylation can amplify neuroinflammatory cell‐death programs in stressed glial cells
	Histone lactylation‐driven FTO pathway [73]	Diabetic retinopathy; endothelial cells	Lactate‐mediated histone lactylation upregulates FTO, stabilizes CDK2 mRNA, and promotes endothelial cell‐cycle progression, tip‐cell formation, inflammation, and microvascular leakage	Histone lactylation couples diabetic metabolic stress to angiogenic cell‐cycle remodeling
	H4K12la in the TRAP1‐HDAC3 axis [74]	Vascular smooth‐muscle‐cell senescence and atherosclerosis	TRAP1‐driven metabolic reprogramming promotes H4K12la, activates SASP‐related transcription, and accelerates VSMC senescence and atherosclerosis	Histone lactylation links mitochondrial/metabolic stress to vascular aging and inflammatory plaque remodeling
	H3K14la‐KLF5 pathway [75]	Diabetic kidney disease	Lactate‐driven H3K14la increases KLF5 expression; KLF5 represses CDH1 and accelerates epithelial–mesenchymal transition and renal fibrosis	Histone lactylation promotes fibrotic epithelial plasticity in diabetic tissue injury
	H4K12laNF‐κB axis [76]	Chronic fatigue syndrome model	PKM2 promotes H4K12la/NF‐κB‐associated neuroinflammation and mitochondrial damage	Histone lactylation can reinforce inflammatory transcription and mitochondrial dysfunction in fatigue‐associated neuroimmune pathology
	H4K12la‐Foxo1 axis [77]	Diabetes‐induced cognitive impairment	H4K12la potentiates mitochondrial oxidative stress through the Foxo1 pathway	Histone lactylation may connect diabetic metabolic stress to neuronal mitochondrial dysfunction and cognitive decline
Non‐histone lactylation non‐histone lactylation non‐histone lactylation	MOESIN K72 lactylation [78]	Tumor‐infiltrating regulatory T cells	Tumor‐derived lactate promotes MOESIN lactylation and enhances TGF‐β signaling in Treg cells	Non‐histone lactylation strengthens Treg‐mediated immunosuppression in lactate‐rich tumors
	PKM2 lactylation [48]	Pro‐inflammatory macrophages	PKM2 lactylation suppresses inflammatory metabolic adaptation and supports reparative macrophage transition	Lactylation can reprogram glycolytic enzymes to restrain inflammatory metabolism and promote repair‐like macrophage states
	HMGB1 lactylation [79]	Polymicrobial sepsis macrophages	Lactate promotes HMGB1 lactylation, acetylation, and exosomal HMGB1 release	Lactylation can promote secretion of inflammatory alarmins during sepsis
	HMGB1 lactylation [80]	Sepsis‐associated acute kidney injury	Macrophage HMGB1 lactylation promotes neutrophil extracellular trap formation through cGAS–STING signaling	Lactylation amplifies innate immune injury by linking macrophage alarmin release to NET‐driven tissue damage
	α‐Myosin heavy chain K1897 lactylation^53^	Heart failure	α‐Myosin lactylation maintains sarcomeric structure and function and alleviates heart failure development	Structural‐protein lactylation can preserve contractile architecture and cardiac function
	Serpina3k/SERPINA3 K351 lactylation [81]	Cardiac ischemia–reperfusion injury	Serpina3k lactylation protects against cardiac I/R injury	Lactylation may stabilize cardioprotective stress‐response proteins during ischemic injury
	p53 K120/K139 lactylation [25]	Tumorigenesis; AARS1‐dependent Lactylation	AARS1 senses lactate and lactylates p53, attenuating p53 DNA binding, transcriptional activity, phase separation, and tumor‐suppressive function	Lactate sensing can directly inhibit tumor‐suppressor function through non‐histone lactylation
	cGAS lactylation [27]	Innate DNA sensing	AARS1/AARS2‐mediated cGAS lactylation suppresses cGAS DNA sensing and innate immune activation	Lactylation can restrain antiviral or anti‐tumor DNA‐sensing pathways
	YAP K90 and TEAD1 K108 lactylation [19]	Gastric cancer	AARS1 promotes YAP/TEAD lactylation, enhancing YAP–TEAD transcriptional activity and tumor‐cell proliferation	Lactylation can activate oncogenic transcription‐factor complexes downstream of lactate sensing
	PDHA1 K336 and CPT2 K457/K458 lactylation [16]	Hypoxia; mitochondrial metabolism	AARS2‐mediated mitochondrial lactylation reduces acetyl‐CoA entry from pyruvate and fatty‐acid oxidation; SIRT3 reverses these marks	Mitochondrial protein lactylation restrains oxidative phosphorylation when lactate accumulation indicates limited oxygen availability
	eEF1A2 K408 lactylation [51]	Colorectal cancer	KAT8‐mediated eEF1A2 lactylation enhances translation elongation and protein synthesis	Lactylation can directly support anabolic protein synthesis in tumor growth
	RPA1 K88 lactylation [82]	Lung adenocarcinoma; radiation response	HAT1‐mediated RPA1 lactylation promotes DNA repair and radioresistance	Lactylation can stabilize genome‐maintenance programs and therapy resistance
	DCBLD1 lactylation [52]	Cervical cancer	DCBLD1 lactylation stabilizes DCBLD1 and activates the pentose phosphate pathway through G6PD	Lactylation supports redox/anabolic metabolism by promoting PPP activation
	YY1 K183 lactylation [83]	Hypoxic retinal microglia/retinal neovascularization	YY1 lactylation enhances YY1 binding to the FGF2 promoter and increases FGF2 transcription, promoting pathological angiogenesis	Lactylation can increase transcription‐factor activity and drive paracrine angiogenic remodeling
	YY1 lactylation [84]	Autoimmune uveitis; activated retinal microglia	YY1 lactylation promotes microglial activation, migration, proliferation, and inflammatory‐gene transcription; p300 inhibition lowers YY1 lactylation and inflammation	Lactylated transcription factors can convert lactate‐rich inflammatory states into amplified immune‐cell activation
	Twist1 K150 lactylation [85]	Hypoxic pulmonary hypertension/endothelial‐to‐mesenchymal transition	Lactate promotes Twist1 lactylation and supports TGF‐β1‐associated EndoMT and vascular remodeling	Lactylation of remodeling transcription factors can stabilize mesenchymal transition programs
	LCP1 lactylation [86]	Cerebral infarction/ischemic injury models	Glycolysis inhibition lowers LCP1 lactylation, promotes LCP1 degradation, and reduces infarct progression and apoptosis	Lactylation may stabilize injury‐promoting cytoskeletal or inflammatory proteins during ischemic damage
	TUFM lactylation [87, 88]	Traumatic brain injury	TUFM lactylation disrupts interaction with TOMM40, impairs mitophagy, and promotes neuronal apoptosis	Lactylation can impair mitochondrial quality control and aggravate neuronal injury
	ATP synthase subunit alpha lactylation [89]	Aortic dissection	Mitochondrial ATP synthase subunit alpha lactylation regulates vascular remodeling and aortic dissection progression	Mitochondrial protein lactylation can regulate vascular remodeling and cardiovascular disease progression
	ALDH2 lactylation [90]	Acute kidney injury	ALDH2 lactylation aggravates mitochondrial dysfunction by disrupting PHB2‐mediated mitophagy	Lactylation can impair mitochondrial quality control in acute tissue injury
	ATP6V1B2 lactylation [91]	Asthma/airway inflammation	Lactate‐driven ATP6V1B2 lactylation impairs V‐ATPase assembly, causes lysosomal dysfunction, and triggers mitochondrial ROS‐dependent pyroptosis	Lactylation connects lactate accumulation to lysosome‐mitochondria dysfunction and inflammatory cell death
	MDH2 lactylation [92]	Renal cancer	KAT8‐mediated MDH2 lactylation enhances mitochondrial function and stress resistance, promoting renal cancer progression	Lactylation of mitochondrial metabolic enzymes can support tumor bioenergetics and survival
	HK2 K346 lactylation [93]	Trophoblast oxidative stress/preeclampsia	Oxidative stress regulates HK2 K346 lactylation, altering HK2 catalytic activity and mitochondrial localization	Lactylation can couple glycolytic enzyme activity and mitochondrial localization to pregnancy‐associated metabolic pathology
	MRE11 lactylation [49]	DNA damage response and drug resistance	MRE11 lactylation promotes homologous recombination repair and contributes to drug resistance	Nuclear non‐histone lactylation can enhance DNA‐repair capacity and therapy resistance
	NBS1 lactylation [50]	DNA repair and chemotherapy resistance	NBS1 lactylation is required for efficient DNA repair and chemotherapy resistance	Lactylation of DNA‐repair machinery links lactate‐rich states to genome maintenance and treatment resistance

### 3.3 Stereochemical Specificity of Lactate‐Derived Protein Modification

In addition to *L*‐Lactate, *D*‐lactate is the less abundant lactate enantiomer, and *D‐*lactylation is a less extensively characterized stereochemical form of lysine lactylation [94] (Table 2). Based on the available evidence, *D*‐lactylation is most clearly linked to bacterial metabolism, glyoxalase‐related chemistry, and selected immune‐regulatory contexts, whereas the mammalian literature remains narrower and more context‐dependent [95, 96, 97].

**TABLE 2:** Distinguishing *D*‐Lactylation From *L*‐Lactylation.

Feature	*L*‐lactate/*L*‐lactylation	*D*‐lactate/*D*‐lactylation	References
Lactate stereoisomer	*L*‐lactate is the predominant lactate enantiomer generated by mammalian glycolysis.	*D*‐lactate is the stereoisomer of *L*‐lactate and normally occurs at much lower concentrations in mammalian tissues and circulation.	[18, 97, 98]
Major metabolic origin in mammals	Primarily generated by reduction of pyruvate through LDHA/LDHB during glycolysis; extracellular *L*‐lactate can also be imported through monocarboxylate transporters.	Endogenously generated mainly through the methylglyoxal–glyoxalase pathway. Methylglyoxal reacts with glutathione and is converted by GLO1 to S‐*D*‐lactoylglutathione (SLG/LGSH), which is subsequently hydrolyzed by GLO2 to *D*‐lactate.	[96, 99, 100]
Contribution of microorganisms	*L*‐lactate is also produced by many microorganisms but is already abundant through host metabolism.	Several intestinal and other microorganisms produce *D*‐lactate; microbial production can substantially increase systemic *D*‐lactate, particularly in short‐bowel syndrome or altered intestinal fermentation.	[101]
Relative abundance in mammals	Dominant lactate species. Concentrations can reach millimolar levels physiologically and rise markedly during intense glycolysis, hypoxia and the Warburg effect.	Normally much less abundant than *L*‐lactate. Substantial accumulation is unusual and is associated particularly with excessive microbial production or impaired *D‐*lactate metabolism.	[101]
Metabolic clearance	Readily interconverted with pyruvate through LDH and participates extensively in metabolic exchange between tissues.	Can be metabolized by mammalian LDHD (*D*‐lactate dehydrogenase); loss‐of‐function variants in LDHD cause elevated *D*‐lactate.	[100]
Immediate lactyl donor	Multiple mechanisms have been reported. *L*‐lactyl‐CoA can serve as an activated donor, whereas AARS1 can use *L*‐lactate + ATP directly to generate an activated lactyl intermediate and transfer the lactyl group to proteins.	In the best‐characterized mammalian pathway, the principal reactive donor is S‐*D*‐lactoylglutathione (SLG/LGSH) rather than free *D*‐lactate itself.	[1, 2, 6, 7, 8, 9, 10, 12, 13, 14, 15, 18, 19, 20, 21, 22, 25, 37, 40, 55, 96, 102]
Relationship between free lactate and lactylation	Relatively direct: increased glycolytic *L*‐lactate strongly promotes *K_L_*_‐la_, and isotope‐labeled *L*‐lactate is incorporated into lactylated proteins.	More indirect in mammals: *D*‐lactate and *K_D_*_‐la_ arise from the same glyoxalase pathway, but SLG is the direct acylating species in the established nonenzymatic mechanism.	[18, 95, 96, 97, 99]
Major formation mechanism	Predominantly regarded as an enzymatically regulated PTM, although several mechanistically distinct routes may coexist.	The best‐established mammalian mechanism is nonenzymatic acyl transfer from SLG to lysine. A nearby cysteine can facilitate transfer through an S‐lactylated intermediate.	[18, 19, 55, 96, 99]
Known or proposed writers	p300/CBP can catalyze lactylation; AARS1 has been demonstrated to act as a direct lactate‐sensing lactyltransferase. ACSS2 can generate lactyl‐CoA and cooperate with KAT2A/GCN5 to catalyse histone lactylation. Additional enzymatic mechanisms continue to emerge.	No dedicated mammalian *D*‐lactyltransferase is firmly established for the SLG pathway because modification can occur nonenzymatically. In *E. coli*, however, *D*‐lactate can be converted to *D*‐lactyl‐CoA by YdiF, demonstrating that enzymatically supported *D*‐lactylation pathways can exist in bacteria.	[18, 19, 55, 95, 96]
Erasers	HDAC1–3 are well‐characterized delactylases; SIRT1–3 also show delactylase activity in vitro, with additional evidence for SIRT2‐dependent delactylation in cells.	HDAC1–3 can remove *K_D_*_‐la_ in biochemical assays, but physiological regulation of *D*‐delactylation remains much less characterized than that of *K_L_*_‐la._	[26]
Histone modification	Well established. Histone *K_L_*_‐la_, including H3K18la and other sites, links glycolytic metabolism to transcriptional regulation.	Can occur on histones, but stereospecific analyzes indicate that *K_D_*_‐la_ is not the dominant glycolysis‐induced histone lactylation species under normal glyoxalase activity.	[18, 26, 98]
Non‐histone modification	Extensively reported on metabolic enzymes, transcription factors, signaling proteins, and structural proteins.	Also occurs broadly on non‐histone proteins. SLG‐driven *D*‐lactylation was recently mapped to thousands of sites in activated macrophages, with substantial enrichment in cytosolic proteins.	[95, 98]
Representative biological function	Links elevated glycolytic flux to chromatin regulation and protein function; reported roles span macrophage polarization, tumor biology, metabolism, signaling and stress adaptation.	Emerging evidence indicates roles in metabolic feedback and immune regulation. *D*‐lactylation of RelA K310, for example, suppresses NF‐κB transcriptional activity and limits excessive inflammatory responses.	[95, 98]
Relationship to glycolysis	Closely follows *L*‐lactate production and is currently considered the principal lactylation isomer responding to glycolysis and the Warburg effect.	Linked to a glycolytic side pathway: increased methylglyoxal/SLG or reduced GLO2 activity can enhance *K_D_*_‐la_. Thus, it reflects glyoxalase metabolism rather than bulk *L*‐lactate production alone.	[95, 97, 98]
Current evidence base	Relatively mature. Supported by isotope tracing, stereospecific antibodies, chemical separation, proteomics, enzymology, and functional studies across numerous mammalian systems.	Emerging and more context‐dependent. Strong biochemical evidence exists for SLG‐dependent modification, with recent studies establishing specific immune and bacterial functions, but considerably fewer systems have been investigated.	[26, 94, 95, 97, 98]

*D*‐lactate is usually described as arising from methylglyoxal/glyoxalase metabolism or microbial pathways rather than as the dominant product of human glycolysis [95, 96, 97, 102]. By contrast, *L*‐lactate is the canonical glycolytic lactate enantiomer and is generally considered the main precursor for the dominant lactylation isomer in eukaryotic systems [18, 98, 99, 103]. The *D*‐lactylation literature also connects *D*‐lactate to bacterial systems, including *Escherichia coli* (*E. coli*), and to infection‐related settings [94, 99, 104, 105].

Mechanistically, *D*‐lactylation is often described as nonenzymatic or indirect, particularly through S‐*D*‐lactoylglutathione‐linked chemistry and glyoxalase II‐related pathways [95, 97]. A separate line of evidence suggests that direct *D*‐lactate‐driven *D*‐lactylation can also occur in bacteria such as *E. coli* [94]. This contrasts with *L*‐lactylation, which is more often presented as the dominant enzymatic or direct lactylation pathway linked to glycolytic *L*‐lactate production, *L*‐lactyl‐CoA, and regulated writer‐associated systems [18, 94, 98, 99].

Several representative substrates have been reported. RelA is a key mammalian immune‐related substrate in S‐*D*‐lactoylglutathione‐induced nonenzymatic *D*‐lactylation, where *D*‐lactylation dampens NF‐κB signaling [95]. In bacteria, *D*‐lactylation has been reported on proteins involved in metabolism and growth control in *E. coli* [95], and lactylation has also been linked to bacterial metabolic regulation and virulence‐associated phenotypes in systems such as *Salmonella* [104, 105]. One infection‐related mammalian disease example is STAT3 K631 lactylation in esophageal squamous cell carcinoma, which has been associated with *D‐*lactate derived from intratumoral *Lactobacillus* [105].

Distinguishing *D*‐lactylation from *L*‐lactylation requires stereochemical discrimination, although the available evidence does not yet provide a single standardized workflow [96, 97]. Mass spectrometry‐based lactylome profiling and site‐confirmation workflows have been used to identify lactylated proteins, but standard lactylome approaches may not always distinguish stereoisomeric lactyl marks without additional stereochemistry‐aware validation [96, 97, 103]. Because *D*‐ and *L*‐lactylation are chemically analogous, isomer‐aware analytical readouts, defined precursor tracing, and orthogonal validation are necessary for rigorous interpretation (Table 3).

**TABLE 3:** Representative Links Between Lactate Signaling and Disease.

Disease/condition	Lactate‐related signaling	Key cellular context	Evidence‐supported downstream effect	Disease implication
**Cancer**	MCT1‐dependent lactate uptake and oxidative metabolism [106]	Oxygenated tumor cells adjacent to hypoxic tumor regions	Lactate uptake fuels mitochondrial respiration and supports metabolic symbiosis between hypoxic lactate‐producing and oxygenated lactate‐consuming tumor cells	Promotes tumor metabolic cooperation and creates vulnerability to MCT1 inhibition
	Lactate‐HIF‐1α signaling in tumor‐associated macrophages [107]	Tumor‐associated macrophages exposed to tumor‐derived lactic acid	Lactic acid induces VEGF and Arg1 expression and promotes M2‐like macrophage polarization	Supports angiogenesis, immune suppression, and tumor growth
	Lactate‐supported Treg metabolism [106]	Tumor‐infiltrating regulatory T cells	Lactic acid supports Treg oxidative metabolism, survival, and suppressive function in glucose‐poor tumors	Reinforces immunosuppressive TME and limits anti‐tumor immunity
	Lactate‐induced PD‐1 expression in Treg cells [23, 61, 62]	Treg cells in highly glycolytic tumors	Lactate increases PD‐1 expression and contributes to Treg functional adaptation in MYC‐amplified and liver tumors	Promotes immune evasion and influences checkpoint‐therapy response
	HCAR1/GPR81 receptor signaling [78]	Colorectal tumor cells	Lactate‐HCAR1 signaling induces CCL2 and CCL7 expression, recruiting immunosuppressive myeloid cells	Promotes tumor immune evasion and may reduce anti‐PD‐1 efficacy
	MOESIN K72 lactylation [78]	Tumor‐infiltrating Treg cells	Lactate promotes MOESIN lactylation, strengthens TGF‐β receptor/SMAD3 signaling, and enhances Treg generation	Enhances Treg‐mediated immune suppression and tumor progression
	H3K18la‐METTL3‐m6A axis [30]	Tumor‐infiltrating myeloid cells	Histone lactylation increases METTL3, enhances RNA m6A modification, and activates JAK1–STAT3 signaling	Promotes myeloid immunosuppression and tumor immune escape
	AARS1‐dependent p53 lactylation [25]	Tumor cells with high lactate/AARS1 activity	AARS1 lactylates p53 and suppresses p53 DNA binding, transcriptional activity, phase separation, and tumor‐suppressive function	Links lactate sensing to tumorigenesis through direct inhibition of p53
	AARS1‐mediated YAP/TEAD lactylation [19]	Gastric cancer cells	Lactate promotes AARS1 nuclear translocation and YAP K90/TEAD1 K108 lactylation, increasing YAP–TEAD transcriptional activity	Drives oncogenic transcription and tumor‐cell proliferation
	H3K14la‐NEDD4‐PTEN axis [67]	Chemotherapy‐resistant hepatocellular carcinoma	H3K14la increases NEDD4 expression; NEDD4 promotes PTEN ubiquitination and degradation, reinforcing glycolysis and drug resistance	Stabilizes chemotherapy‐resistant metabolic states
	H3K18la‐RUBCNL/Pacer axis [68]	Colorectal cancer under bevacizumab pressure	Tumor‐derived lactate increases H3K18la and RUBCNL expression, promoting autophagosome maturation	Supports autophagy‐mediated bevacizumab resistance
**Metabolic disorders**	HCAR1/GPR81 receptor signaling [108]	Adipocytes	Lactate directly activates GPR81/HCAR1 and suppresses adipose lipolysis	Regulates free‐fatty‐acid release and may influence obesity/insulin‐resistance physiology
	Adipocyte‐derived lactate‐PHD2 signaling [109]	Adipose‐tissue macrophages in obesity	Adipocyte‐derived lactate potentiates adipose macrophage inflammation by targeting PHD2	Links obese adipose glycolytic stress to macrophage inflammation and metabolic dysfunction
	Lactylation‐driven FTO‐CDK2 pathway [73]	Diabetic retinopathy; retinal endothelial cells	Lactate‐mediated histone lactylation upregulates FTO, stabilizes CDK2 mRNA, and promotes endothelial cell‐cycle progression, tip‐cell formation, inflammation, and leakage	Drives diabetic microvascular anomalies and pathological angiogenesis
	H3K14la‐KLF5 pathway [110]	Diabetic kidney disease; renal tubular epithelial cells	Lactate‐driven H3K14la increases KLF5 expression; KLF5 represses CDH1 and promotes epithelial–mesenchymal transition	Promotes renal fibrosis and DKD progression
	H4K12la‐Foxo1 axis [77]	Diabetes‐induced cognitive impairment	H4K12la potentiates mitochondrial oxidative stress through Foxo1‐related signaling	Links diabetic metabolic stress to neuronal mitochondrial dysfunction and cognitive decline
	HK2 K346 lactylation [93]	Trophoblast oxidative stress/preeclampsia	Oxidative stress regulates HK2 K346 lactylation, altering HK2 catalytic activity and mitochondrial localization	Connects glycolytic enzyme lactylation to pregnancy‐associated metabolic pathology
**Inflammatory disease**	Histone lactylation in macrophages [18]	Bacterially challenged M1 macrophages	Late‐phase histone lactylation induces homeostatic and wound‐healing genes such as Arg1	Provides a metabolic ‘lactate clock’ that can shift macrophages toward reparative programs after inflammation
	Lactate‐MAVS binding [20]	RIG‐I‐like receptor antiviral signaling; mitochondrial‐associated membranes	Lactate binds MAVS, prevents MAVS aggregation, and suppresses type I interferon signaling	Dampens antiviral innate immune activation in lactate‐rich states
	HMGB1 lactylation and acetylation^79^	Macrophages during polymicrobial sepsis	Lactate promotes HMGB1 lactylation, acetylation, and exosomal HMGB1 release	Amplifies inflammatory alarmin release and sepsis‐associated immune injury
	HMGB1 lactylation‐cGAS/STING‐NET axis [80]	Macrophages and neutrophils in sepsis‐associated acute kidney injury	Lactate‐induced macrophage HMGB1 lactylation promotes neutrophil extracellular trap formation through cGAS/STING signaling	Links macrophage lactylation to NET‐driven renal injury in sepsis
	H3K18la‐NOD2 axis [72]	Astrocytes in bilirubin encephalopathy	H3K18la increases NOD2 expression, activates MAPK/NF‐κB signaling, and promotes astrocyte pyroptosis	Drives neuroinflammatory cell death in bilirubin‐induced brain injury
	YY1 lactylation [84]	Activated retinal microglia in autoimmune uveitis	YY1 lactylation promotes microglial activation, migration, proliferation, and inflammatory‐gene transcription	Converts lactate‐rich inflammatory niches into amplified ocular inflammation
	ATP6V1B2 lactylation [91]	Airway inflammation/asthma	Lactate‐driven ATP6V1B2 lactylation impairs V‐ATPase assembly, causes lysosomal dysfunction, and triggers mitochondrial ROS‐dependent pyroptosis	Promotes inflammatory cell death and asthmatic airway inflammation
**Cardiovascular disease**	Histone lactylation in monocytes/macrophages [46]	Monocytes after myocardial infarction	Histone lactylation activates reparative genes and supports anti‐inflammatory/pro‐angiogenic monocyte–macrophage programs	Promotes cardiac repair after myocardial infarction
	α‐MHC K1897 lactylation 53[]	Cardiomyocytes/sarcomere	α‐myosin heavy‐chain lactylation maintains sarcomeric structure and function	Protects against heart‐failure development
	Serpina3k lactylation [81]	Cardiac ischemia‐reperfusion injury	Serpina3k lactylation protects against cardiac ischemia–reperfusion injury	Suggests cardioprotective non‐histone lactylation during acute ischemic stress
	Mitochondrial ATP synthase subunit alpha lactylation [89]	Vascular smooth‐muscle remodeling/aortic dissection	Lactylation of mitochondrial ATP synthase subunit alpha regulates mitochondrial function, vascular remodeling, and disease progression	Promotes or marks pathological remodeling in aortic dissection
	Mitochondrial protein lactylome remodeling [111]	Myocardial infarction	MCT1 modulation reshapes the mitochondrial lactylome, reduces mitochondrial ROS, improves bioenergetics, and attenuates inflammatory/fibrotic remodeling	Links mitochondrial lactylation to post‐MI metabolic recovery and remodeling
	H4K12la in TRAP1HDAC3 axis [74]	Vascular smooth‐muscle‐cell senescence and atherosclerosis	TRAP1‐driven metabolic reprogramming promotes H4K12la and activates SASP‐related transcription	Links metabolic stress to VSMC senescence and atherosclerotic plaque remodeling

## 4 Compartmental Lactate and Lactate Signaling

A compartment‐oriented perspective provides a useful way to organize lactate‐associated signaling mechanisms across different cellular and tissue contexts. The preceding sections described how lactate is generated, transported, and sensed. Here, we use extracellular, cytosolic, mitochondrial‐associated, and nuclear compartments as an organizational framework to illustrate how the biological meaning of lactate depends on local exposure, transporter expression, metabolic state, pH, redox environment, and the availability of lactate‐responsive proteins or lactylation machinery. This framework is intended to synthesize existing evidence rather than to establish lactate as a universal regulator of the catabolism‐anabolism axis.

### 4.1 Extracellular Lactate

Extracellular lactate arises when glycolytically active cells export lactate together with protons through monocarboxylate transporters, both under aerobic and hypoxic conditions. MCT4‐dependent lactate export from glycolytic cells and MCT1‐dependent lactate uptake by oxidative cells allow lactate to function as both a metabolic substrate and a spatial signal [3, 10]. In the tumor microenvironment, MCT4‐mediated export from hypoxic regions and MCT1‐dependent uptake by oxygenated cells establish metabolic symbiosis, supporting tumor growth and oxidative metabolism [9, 10]. MCT‐mediated lactate access directly influences immune function. For example, MCT1 blockade rescued dendritic‐cell subsets from tumor‐induced metabolic skewing and restored anti‐tumor T cell fitness, indicating that lactate access can directly influence antigen‐presenting‐cell function [112]. In vivo isotope‐tracing studies further show that lactate can serve as a major carbon source for the TCA cycle in tumors and normal tissues, supporting the concept that lactate is a circulating and locally exchanged metabolic substrate [6, 7]. Physiological lactate transfer is also evident in the nervous system, where astrocyte‐neuron lactate transport supports neuronal function and long‐term memory formation [12, 13]. Thus, MCTs may determine whether a cell can interpret lactate as fuel, acid‐base input, redox input, or signaling substrate. By doing so, they actively shape tissue ecology and intercellular communication.

MCTs regulate lactate flux and metabolic availability, whereas HCAR1 serves as a non‐transporting receptor that detects extracellular lactate and propagates signaling cascades. In breast cancer, GPR81 supports tumor‐cell survival and metabolic adaptation, promotes cancer growth through autocrine and paracrine mechanisms, and regulates breast cancer glycolysis and tumor growth. HCAR1 activation induces CCL2 and CCL7 expression in colorectal tumor cells, recruits immunosuppressive CCR2^+^ PMN‐MDSCs, weakens CD8^+^ T cell‐mediated antitumor immunity, and reduces responsiveness to immune checkpoint therapy [36]. HCAR1 also functions in the nervous system, where it modulates neuronal network activity and participates in lactate‐dependent vascular or injury responses. Therefore, HCAR1 provides a receptor‐based layer through which extracellular lactate gradients are converted into signaling outputs.

Transporter‐dependent lactate uptake and extracellular lactic acid reshape immune‐cell function in a strongly context‐dependent manner. Tumor‐derived lactic acid suppresses human cytotoxic T lymphocyte proliferation, cytokine production, and cytotoxicity, while LDHA‐dependent lactic acid production blunts T‐cell and NK‐cell immunosurveillance [14, 15]. Tumor‐derived lactic acid polarizes tumor‐associated macrophages toward a tumor‐promoting state through HIF‐1α‐dependent induction of VEGF and Arg1 in syngeneic murine tumor models of Lewis lung carcinoma and B16‐F1 (B16) melanoma cancer cell lines [113]. Regulatory T cells can metabolically adapt to lactate‐rich tumors and use lactic acid to maintain suppressive function and proliferation; in highly glycolytic tumors, lactic acid promotes NFAT1 nuclear translocation and PD‐1 expression in Treg cells [23]. Lactate can also attenuate dendritic‐cell IL‐12 production and reduce immune checkpoint inhibitor efficacy [114]. However, lactate is not uniformly immunosuppressive. Sodium lactate can increase stem‐like TCF‐1^+^ CD8^+^ T cells and augment antitumor immunity under defined experimental conditions [23]. In solid tumors, lactic acid can suppress CD8^+^ T‐cell activation through the SENP1‐MondoA‐TXNIP axis, while in AML bone marrow, elevated lactate contributes to tumor‐associated macrophage polarization, suggesting that lactate‐driven immune suppression is not restricted to solid tumors [115, 116, 117]. Lactate can also support innate immune memory. Under physiologically relevant carbon‐source conditions, trained monocytes preferentially use lactate as a TCA‐cycle substrate, and lactate metabolism supports trained immunity by promoting cytokine responses, chromatin accessibility, and histone lactylation. These findings show that lactate signaling depends on lactate form, pH, transporter expression, nutrient availability, exposure duration, and immune‐cell state (Figure 2A).

**FIGURE 2:**
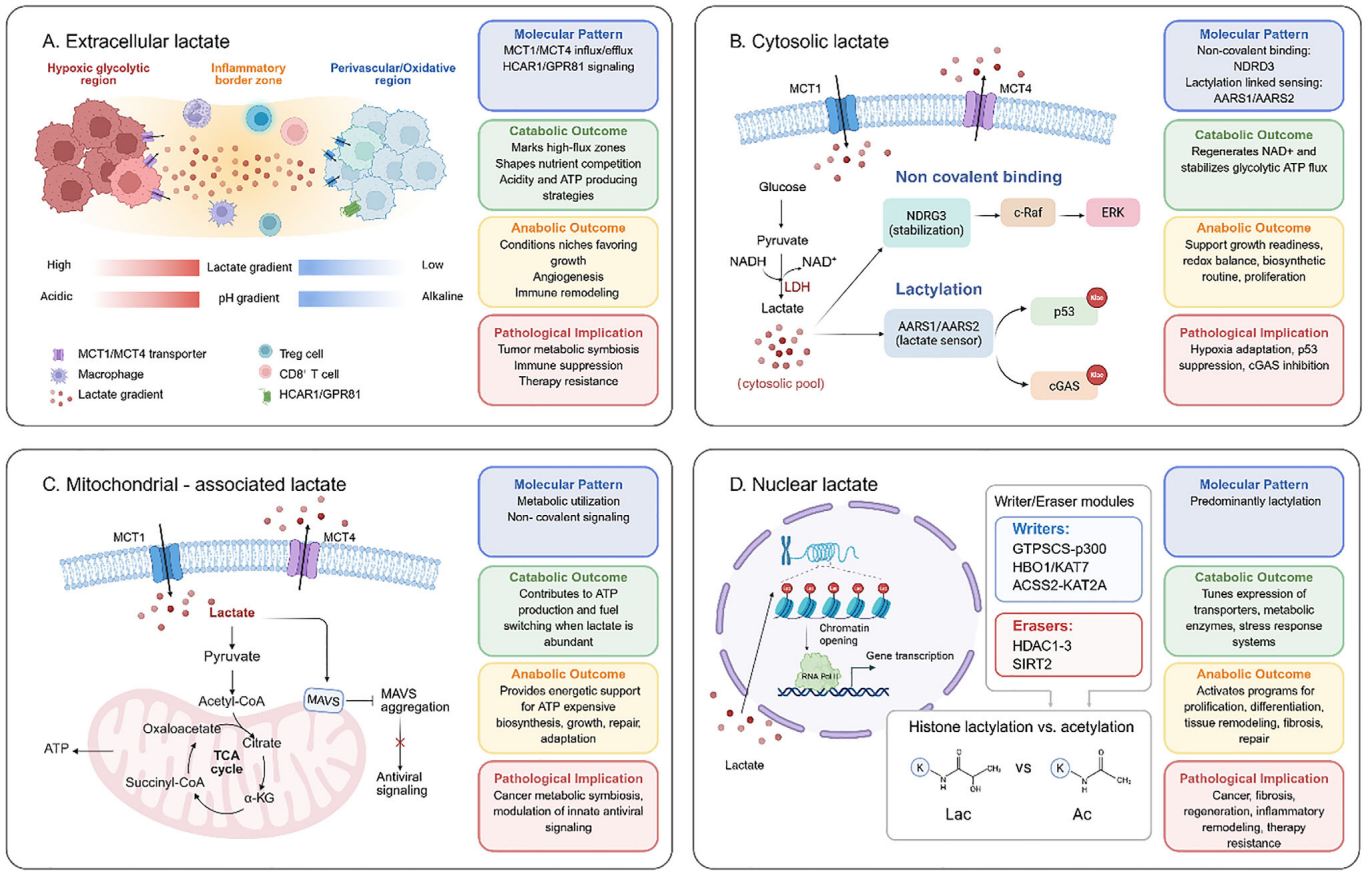
Compartmental organization of lactate‐associated signaling and protein lactylation. Lactate‐associated signaling is shaped by compartmental exposure and molecular interpretation. In the extracellular space, lactate gradients generated by glycolytic cells can influence recipient cells through MCT‐mediated transport and HCAR1/GPR81‐dependent receptor sensing. In the cytosol, lactate is linked to LDH‐dependent redox balance, transporter‐mediated flux, and direct regulation of lactate‐responsive proteins such as NDRG3 and MAVS. In mitochondrial‐associated contexts, lactate‐derived carbon can support oxidative metabolism after conversion to pyruvate, whereas mitochondrial protein lactylation can modulate oxidative phosphorylation, redox balance, and mitochondrial quality control. In the nucleus, lactate‐associated metabolism can be translated into histone and non‐histone lactylation, including ACSS2‐KAT2A, GTPSCS‐p300, and AARS‐dependent pathways. Together, these mechanisms illustrate how lactate‐rich states can influence metabolism, signaling, chromatin regulation, immune responses, tissue remodeling, and disease progression in a context‐dependent manner. MCT, monocarboxylate transporter; HCAR1, hydroxycarboxylic acid receptor 1; LDH, lactate dehydrogenase; GTPSCS, GTP‐specific succinyl‐CoA synthetase; Kla, lysine lactylation. Figure created using BioRender.com.

### 4.2 Cytosolic Lactate

Cytosolic lactate is generated primarily by lactate dehydrogenase, which converts pyruvate to lactate while oxidizing NADH to NAD^+^, thereby sustaining glycolytic flux and ATP production under high energetic demand. The size and behavior of this cytosolic pool are shaped by LDH activity, MCT‐mediated transport, lactate/pyruvate balance, and cytosolic redox state. In this compartment, the primary catabolic function of cytosolic lactate production is to regenerate NAD^+^, allowing glycolysis to continue when mitochondrial oxidation is constrained or when biosynthetic demands are high. This redox‐linked role positions cytosolic lactate as a metabolic node that connects glycolytic flux, transporter activity, and downstream signaling or lactylation pathways, rather than merely a byproduct of glycolysis. The cytosolic pool interfaces with tumor‐intrinsic metabolic reprogramming and immune‐checkpoint control. ACVR2A attenuation in hepatocellular carcinoma increases lactate‐producing hyperglycolysis, whereas PRMT3‐dependent PDHK1 activation increases lactate production and drives H3K18la‐dependent PD‐L1 transcription, illustrating how cytosolic lactate production can be translated into extracellular immune suppression and nuclear checkpoint‐gene regulation [118, 119]. LDHA‐dependent lactate production can also support glycolytic metabolism while simultaneously suppressing anti‐tumor immunity through extracellular lactic acid accumulation [15].

A high cytosolic lactate concentration marks a high‐flux glycolytic state that can be interpreted by intracellular sensors to promote growth and adaptation. For example, accumulated lactate stabilizes NDRG3 under prolonged hypoxia, activating the c‐Raf‐ERK pathway to drive angiogenesis and cell growth [17]. Similarly, cytosolic lactate directly binds MAVS at mitochondrial‐associated membranes, suppressing antiviral type I interferon signaling as described in Section 3.1 [20]. These non‐covalent sensing mechanisms provide direct routes from cytosolic lactate accumulation‐a catabolic signature‐to anabolic transcriptional and adaptive programs.

Furthermore, beyond its roles in redox balance and signaling, cytosolic lactate serves as a precursor pool for covalent modifications. The cytosolic availability of lactate or its derivative, lactyl‐CoA, feeds into the nuclear and mitochondrial lactylation machinery described in subsequent sections. This positioning makes the cytosol a critical hub where glycolytic flux is not only used for acute energy production but also transduced into signals that regulate proliferation, immune evasion, and hypoxia adaptation (Figure 2B).

### 4.3 Mitochondrial‐Associated Lactate Utilization, Lactate Redox Handling, and Mitochondrial Protein Lactylation

Lactate is a major oxidative carbon source, and mitochondrial disposal is a central endpoint of the intracellular and cell‐cell Lactate Shuttles [2, 3, 5, 7, 120]. In situ preparations that preserve the native mitochondrial reticulum in human skeletal muscle demonstrate lactate‐supported respiration and provide direct replication of earlier rodent observations in human tissue [121]. Consistent with these biochemical findings, stable‐isotope studies show extensive entry of circulating lactate carbon into the tissue TCA cycle [6, 7], and hyperpolarized ^13^C MRI has visualized lactate‐to‐pyruvate conversion followed by oxidation in the living human brain [122]. These data support a mitochondria‐associated lactate oxidation system involving transport, LDH‐dependent conversion, and respiratory disposal across the reticulum [5, 119, 121, 123]. Mechanistically, lactate‐derived carbon is oxidized by mitochondria after conversion to pyruvate. Mitochondrial‐associated lactate handling also intersects with cellular redox and signaling pathways. Lactate can directly suppress MAVS aggregation and type I interferon production [20], providing an example of non‐ covalent lactate signaling at mitochondria‐associated membranes, while mitochondrial lactate venting can limit oxidative stress. These findings position mitochondrial‐associated lactate as both an oxidative fuel pathway and a regulator of redox and innate immune signaling.

Recent studies extend this framework to mitochondrial protein lactylation. Under hypoxia, AARS2 accumulates and catalyzes mitochondrial lactylation of PDHA1 at K336 and CPT2 at K457/K458, thereby reducing acetyl‐CoA influx from pyruvate and fatty‐acid oxidation, whereas SIRT3 removes these lactylation marks to restore oxidative phosphorylation [16]. This finding indicates that mitochondrial lactylation can restrain ATP‐generating catabolic pathways when hypoxia and lactate accumulation signal limited oxidative capacity. Lactylation of mitochondrial ATP synthase subunit alpha regulates vascular remodeling and disease progression, suggesting that SIRT3‐linked mitochondrial protein lactylation can operate in cardiovascular pathology [16, 59, 111]. Further, quantitative mitochondrial lactylome profiling revealed broad remodeling of mitochondrial protein lactylation across bioenergetic, redox, and metabolic pathways; modulation of MCT1 reshaped this lactylome, reduced mitochondrial reactive oxygen species, improved mitochondrial bioenergetics, and attenuated inflammatory or fibrotic remodeling [111]. Together, these studies suggest that mitochondrial lactylation is not uniformly damaging or protective, but instead acts as a context‐dependent regulator of mitochondrial metabolism, redox balance, and tissue remodeling.

Disease‐focused studies further connect mitochondrial lactylation to pregnancy complications, acute kidney injury, airway inflammation, mitochondrial diseases, and cancer metabolism. In trophoblasts, oxidative stress regulates HK2 K346 lactylation, altering HK2 catalytic activity and mitochondrial localization, thereby linking lactate‐dependent protein modification to mitochondrial binding, glycolytic flux, and the pathogenesis of preeclampsia [93]. In acute kidney injury, ALDH2 lactylation aggravates mitochondrial dysfunction by disrupting PHB2‐mediated mitophagy, indicating that lactylation can impair mitochondrial quality control [90]. In asthma, lactate‐driven ATP6V1B2 lactylation impairs V‐ATPase assembly, causes lysosomal dysfunction, triggers mitochondrial ROS‐dependent pyroptosis, and promotes airway inflammation [91]. Single‐cell and bulk transcriptomic analyses of mitochondrial diseases implicate lactylation‐associated programs in disease‐relevant metabolic and cellular states, supporting the need to consider lactylation as part of mitochondrial disease biology [124]. In renal cancer, KAT8‐mediated lactylation of mitochondrial malate dehydrogenase 2 promotes tumor progression by enhancing mitochondrial function and stress resistance, providing a direct example in which mitochondrial enzyme lactylation supports tumor bioenergetics and survival [92]. Collectively, these findings broaden mitochondrial lactylation from a hypoxia‐adaptation mechanism to a disease‐associated regulatory layer that can influence oxidative phosphorylation, redox control, mitochondrial quality control, stress tolerance, inflammatory cell death, cardiovascular remodeling, and cancer progression.

Together, these studies demonstrate that mitochondrial‐associated lactate contributes to ATP production and fuel switching when lactate is abundant and oxidative capacity is available, while mitochondrial lactylation can limit or redirect oxidative flux depending on hypoxia, redox state, and disease context. Anabolically, lactate‐supported mitochondrial metabolism can provide energy, carbon flow, and metabolic flexibility for proliferation, tissue repair, immune remodeling, or tumor adaptation, whereas mitochondrial or mitochondria‐connected lactylation can stabilize disease‐associated states by tuning enzyme activity, oxidative stress resistance, mitophagy, and inflammatory remodeling (Figure 2C).

### 4.4 Nuclear Lactate

Nuclear lactate signaling is most clearly represented by histone lysine lactylation, a lactate‐derived chromatin modification that links glycolytic metabolism to transcriptional regulation [18]. Genome‐wide profiling further showed that H3K18 lactylation marks tissue‐specific active enhancers across multiple cell and tissue contexts, supporting the view that lactylation is not only a global lactate‐associated mark but also a spatially organized chromatin signal [64]. In ischemic or injured tissues, histone lactylation has also been linked to reparative gene activation after myocardial infarction and to macrophage gene‐expression dynamics during ischemia‐induced muscle regeneration [46, 125]. In development and cell‐state transition, glycolysis‐regulated histone lactylation couples metabolic state to developmental gene‐regulatory networks, and Dux‐induced H3K18la promotes early iPSC reprogramming by recruiting Brg1 as a histone‐lactylation reader [65, 66, 126]. Long‐term H3K18 lactylation connects metabolic rewiring to trained immunity after BCG vaccination [126]. In small‐cell lung cancer, lactate‐induced H3K18la promotes Nur77‐dependent immune escape 127. PRMT3‐PDHK1‐driven lactate production enhances H3K18la occupancy at the PD‐L1 promoter, while in lung cancer PD‐L1 K280 lactylation stabilizes PD‐L1 protein itself, showing that lactate can regulate immune checkpoints at both chromatin and protein‐stability levels [62, 118]. Together, these studies indicate that nuclear lactylation can encode lactate‐rich metabolic states into enhancer activity, transcriptional remodeling, tissue repair, and developmental plasticity.

The nuclear lactylation machinery includes writers, erasers, and emerging readers. The original histone lactylation study implicated p300 as an early lactylation writer, while later work identified HBO1/KAT7 as a lysine lactyltransferase that mediates H3K9la‐dependent transcription [54]. Recent work has identified the GTPSCS‐p300 axis as a compartment‐specific mechanism linking lactate metabolism to histone lactylation. Nuclear GTPSCS is composed of the common alpha subunit SUCLG1 and the GTP‐specific beta subunit SUCLG2. This SUCLG1‐SUCLG2 complex was reported to interact with p300 and function as a lactyl‐CoA synthetase, thereby generating lactyl‐CoA locally for p300‐mediated histone lysine lactylation [58, 128]. In glioma models, this pathway increased histone lactylation, particularly H3K18la, and promoted oncogenic transcriptional programs and glioma progression without similarly increasing histone succinylation. ACSS2 can also function as a lactyl‐CoA synthetase and couple with KAT2A to promote histone lactylation and tumor immune evasion [55]. Histone lactylation is further interpreted by reader proteins:Brg1 recognizes H3K18la during reprogramming, whereas DPF2 reads H3K14la to drive transcription and tumorigenesis [66, 129]. These writer–eraser–reader studies support the concept that nuclear lactylation is a regulated metabolic–epigenetic system rather than a passive chemical footprint of lactate accumulation [130].

Nuclear lactate‐associated regulation also extends to non‐histone proteins that control tumor suppression, transcription, and genome maintenance. AARS1 senses lactate and catalyzes p53 lactylation, which attenuates p53 DNA‐binding affinity [25]. DNA‐repair proteins provide another major nuclear target class: MRE11 lactylation promotes homologous recombination repair and drug resistance, while NBS1 lactylation is required for efficient DNA repair and chemotherapy resistance [49, 50]. HAT1‐mediated RPA1 lactylation has also been linked to DNA repair and radioresistance in lung adenocarcinoma [82]. Thus, nuclear lactylation links lactate‐rich metabolic states to transcriptional activation, chromatin accessibility, developmental remodeling, tumor immune evasion, DNA repair, and therapy resistance (Figure 2D).

## 5 Emerging Evidence of Lactate Signaling in Disease Initiation, Progression, and Therapeutic Response

The disease relevance of lactate signaling extends well beyond its accumulation as a metabolic by‐product. In pathological tissues, elevated lactate generally reflects broader changes in glycolytic flux, oxygen availability, nutrient competition, inflammatory tone, vascular perfusion, stromal remodeling, and cellular stress. Lactate‐rich environments can therefore influence disease biology at multiple stages, including disease initiation, progression, and therapeutic response [131, 132]. Importantly, lactate does not act through a single linear pathway. Its effects arise from the combined actions of transporter‐mediated metabolic exchange, receptor‐dependent sensing, direct protein interactions, mitochondrial metabolic adaptation, and covalent protein modification through lactylation [6, 7].

During disease initiation, lactate signaling is unlikely to act as an isolated primary cause. Instead, lactate‐rich states may create permissive environments that facilitate adaptation to hypoxia, inflammation, nutrient limitation, oxidative stress, infection, or repeated tissue injury. In premalignant or chronically inflamed tissues, increased glycolytic flux and local lactate accumulation can enhance cell survival, alter immune activation, reshape macrophage and stromal states, and influence transcription through histone lactylation [75, 107, 113, 133, 134]. In metabolic and vascular diseases, lactate‐associated signaling may similarly represent an early response to tissue stress, mitochondrial dysfunction, or altered substrate utilization [50, 65, 75]. Thus, lactate is more likely to contribute to disease initiation by reinforcing early adaptive, inflammatory, or stress‐response programs than by acting as a universal initiating lesion.

The contribution of lactate becomes more evident during disease progression, when persistent lactate‐rich environments can stabilize pathological cellular states and coordinate interactions among multiple cell populations. In cancer, lactate supports metabolic cooperation between hypoxic glycolytic cells and oxidative recipient cells, contributes to extracellular acidification, promotes angiogenesis and stromal remodeling, and shapes immunosuppressive myeloid and T cell programs [6, 10, 14, 106, 112, 135, 136]. HCAR1/GPR81 signaling, MCT‐mediated lactate exchange, and lactylation further connect tumor metabolism to immune evasion, transcriptional remodeling, DNA repair, and therapeutic resistance [41, 63, 78, 80, 82]. Similar mechanisms operate outside cancer. In inflammatory and fibrotic diseases, lactate can regulate macrophage polarization, fibroblast or stellate‐cell activation, endothelial remodeling, and tissue‐repair programs [29, 80, 86, 107]. In cardiovascular, neurological, renal, and metabolic disorders, lactate‐associated signaling can influence mitochondrial metabolism, redox balance, cell death, vascular remodeling, and inflammatory injury [73, 74, 75, 81, 86, 125]. Collectively, these observations indicate that lactate contributes to disease progression not simply as a fuel, but as a context‐dependent signal that links metabolic state to intercellular communication and tissue remodeling.

Lactylation provides a mechanism by which lactate‐rich disease states may be translated into dynamic but potentially longer‐lasting regulatory outputs. Histone lactylation can link altered metabolism to transcriptional programs involved in inflammation, tissue repair, fibrosis, cancer progression, and immune regulation, whereas non‐histone lactylation may directly modify enzymes, transcription factors, DNA‐repair proteins, immune regulators, mitochondrial proteins, and structural proteins [16, 18, 26, 29, 30]. For example, lactylation of DNA‐repair proteins can promote genome maintenance and treatment resistance, lactylation of immune or checkpoint‐related proteins can reinforce immune evasion, and mitochondrial protein lactylation can modulate oxidative phosphorylation, redox balance, or mitochondrial quality control. However, not every reported lactylation event should be interpreted as functionally causal. A key challenge for disease studies is to distinguish lactylation as a marker of altered metabolic state from lactylation as a driver of pathological phenotype.

Lactate signaling also has important implications for therapeutic response. In tumors, lactate‐rich microenvironments can limit cytotoxic T‐cell and natural killer‐cell function, promote suppressive myeloid or regulatory T‐cell states, and reduce responsiveness to immune checkpoint blockade [14, 15, 23, 41, 82, 135]. Lactate‐associated extracellular acidification, mitochondrial adaptation, DNA repair, and lactylation‐dependent transcriptional programs may also influence responses to chemotherapy, radiotherapy, targeted therapy, anti‐angiogenic therapy, and metabolic intervention [61, 137, 138, 139]. In non‐malignant diseases, lactate‐targeted strategies require even greater caution because lactate can be either detrimental or protective depending on timing, tissue context, and cell type. For example, lactate utilization may support energetic recovery and tissue repair in some cardiovascular or regenerative settings, whereas excessive lactate accumulation or lactylation may promote inflammation, fibrosis, vascular remodeling, or mitochondrial dysfunction in others [29, 46, 53, 140, 141].

Overall, lactate signaling should be viewed as a disease‐modifying network rather than a single pathogenic pathway (Figure 3). Its biological outcome depends on the source of lactate, the responding cell type, local oxygen and nutrient availability, transporter and receptor expression, pH, redox state, exposure duration, and the capacity of cells to convert lactate‐associated metabolic states into covalent protein modifications. This framework helps explain why lactate can promote tumor progression and immune suppression in some contexts while supporting tissue repair, metabolic adaptation, or stress tolerance in others.

**FIGURE 3:**
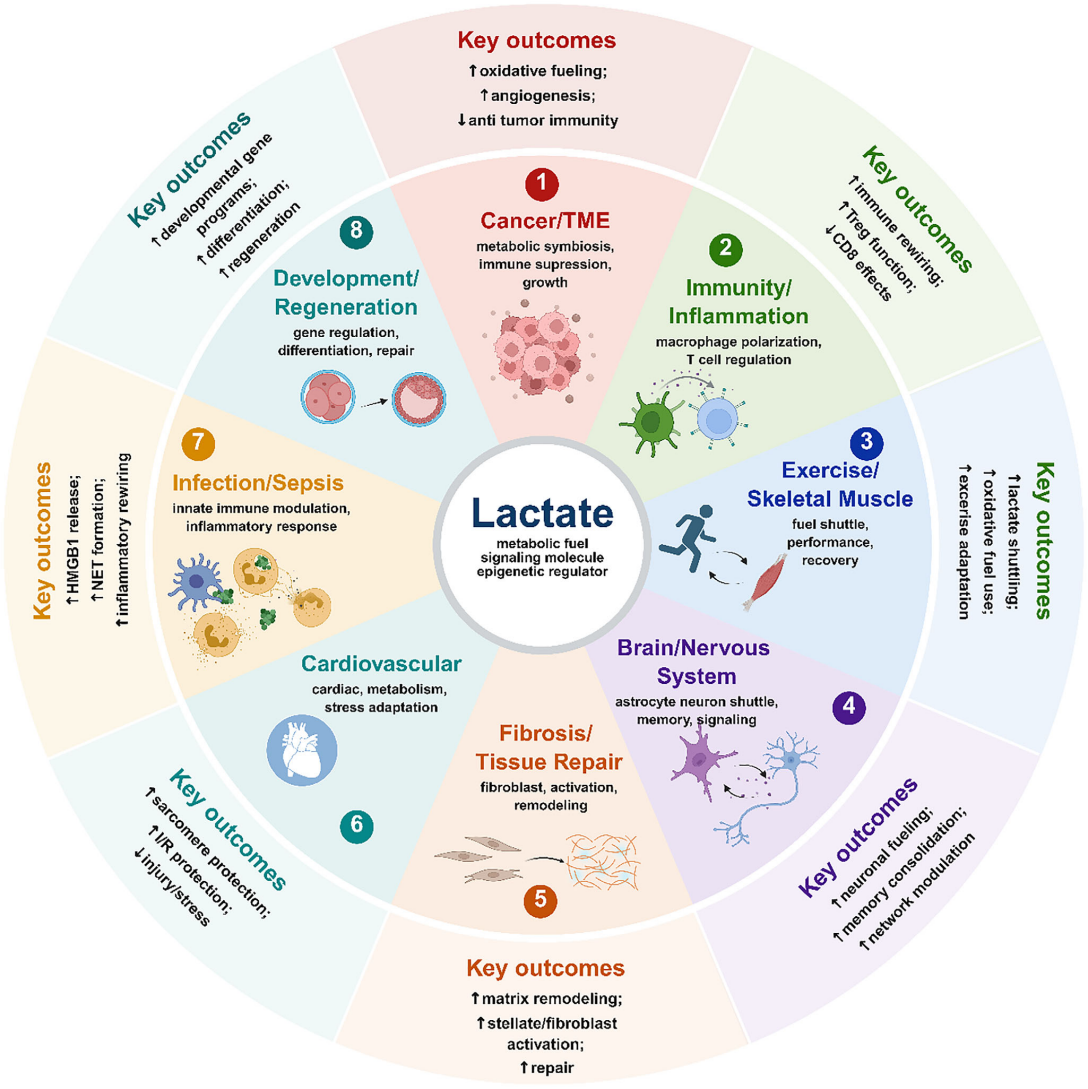
Context‐dependent roles of lactate in physiology and disease. Lactate functions as a metabolic fuel, signaling metabolite, and epigenetic regulator whose effects vary across biological contexts. In cancer and the tumor microenvironment (TME), lactate supports oxidative fuel use by recipient cells, angiogenesis, and immune evasion. In immune and inflammatory settings, lactate can rewire macrophage and T cell states, promote regulatory T cell function, and exert context‐dependent effects on CD8^+^ T cell responses. During exercise, lactate shuttling supports oxidative fuel use and metabolic adaptation in skeletal muscle. In the nervous system, astrocyte‐neuron lactate exchange contributes to neuronal fueling, memory consolidation, and network modulation. Lactate‐associated signaling also contributes to matrix remodeling and fibroblast or stellate‐cell activation during fibrosis and tissue repair, while in cardiovascular contexts, lactate‐linked pathways can support sarcomere integrity, protection from ischemia‐reperfusion injury, and stress adaptation. During infection and sepsis, lactate‐related mechanisms regulate inflammatory outputs, including HMGB1 release and neutrophil extracellular trap formation. In development and regeneration, lactate‐linked metabolic and epigenetic programs contribute to gene regulation, differentiation, and repair. Figure created using BioRender.com.

## 6 Conclusion and Perspectives

Lactate is now recognized as more than a simple glycolytic by‐product. Current evidence supports its roles as a mobile carbon source, redox‐linked metabolite, receptor ligand, protein‐regulatory molecule, and precursor or substrate for lactylation‐associated protein modification. In this Review, we used a compartment‐oriented framework to integrate these independently reported mechanisms across extracellular, cytosolic, mitochondrial‐associated, and nuclear contexts. This framework should be viewed as a conceptual synthesis rather than a definitive signaling paradigm. Its value lies in clarifying how lactate production, transport, local exposure, molecular sensing, and lactylation can act together in a context‐dependent manner. Two molecular patterns are central to this function. Non‐covalent lactate binding and sensing provide rapid, reversible mechanisms by which lactate can regulate receptors, signaling proteins, and stress‐response pathways. Lactylation provides a covalent mechanism by which lactate‐associated metabolic states can be encoded into chromatin and the proteome, potentially generating longer‐lasting effects on transcription, protein function, and cellular identity. Together, these mechanisms help explain how lactate can act as a fuel, receptor ligand, direct protein‐regulatory molecule, stress‐associated metabolite, immune modulator, and precursor or substrate for covalent protein modification in a context‐dependent manner.

Several important questions remain unresolved. First, how do cells distinguish lactate as a fuel from lactate as a signal? This likely depends on compartmental concentration, transporter expression, pH, redox state, and availability of lactate‐sensitive proteins. Second, what determines whether lactate promotes adaptive repair versus pathological growth or immune dysfunction? The answer may lie in exposure duration, cell type, and tissue architecture. Third, how are non‐covalent lactate sensing and lactylation coordinated? These mechanisms are often studied separately, but in living tissues they may act together to convert transient metabolic gradients into durable biological outcomes.

For translational application, lactate signaling should be targeted with spatial precision. Blocking lactate production alone may not be sufficient, because lactate can be produced, transported, consumed, or interpreted differently across tissue compartments. Therapeutic strategies targeting lactate transporters, lactate receptors, lactate‐binding proteins, or lactylation‐related enzymes will require careful attention to where lactate is produced, where it accumulates, and which cells depend on lactate signaling. In this sense, the future of lactate biology is not only biochemical but also spatial; understanding lactate signaling requires mapping lactate flux, compartmental exposure, and molecular interpretation together.

**BOX 1 Histone lactylation versus acetylation**

Histone lactylation and acetylation are related lysine acyl modifications, but primary research supports the view that they encode partially distinct metabolic inputs. Histone acetylation is classically tied to acetyl‐CoA availability, whereas histone lactylation reflects lactate‐rich states generated by glycolysis, hypoxia, inflammation, exogenous lactate exposure, or lactyl‐CoA/lactate‐AMP‐dependent writer systems [18, 25, 55]. Recent studies further show that histone lactylation can directly couple glycolytic lactate production to immune checkpoint and transcription‐factor programs. In small‐cell lung cancer, an LDH‐H3K18la‐Nur77 axis potentiates immune escape via histone lactylation at H3K18la that induces transcriptional activation of Nur77 in naïve CD8^+^ T cells, leading to tonic TCR signaling, whereas PRMT3 methylates PDHK1, increases lactate production, and promotes H3K18la binding at the PD‐L1 promoter to enhance PD‐L1 expression and immune evasion [118]. Long‐term BCG‐induced trained immunity is likewise associated with persistent H3K18 lactylation in vivo, positioning lactylation as a durable epigenetic mark of innate immune memory [127]. During macrophage polarization, histone lactylation shows temporal dynamics distinct from acetylation and is associated with late‐phase homeostatic and wound‐healing gene expression. In hepatic stellate cells, HK2 catalytic activity alters gene expression via histone lactylation rather than histone acetylation, supporting the idea that lactylation can encode a lactate‐specific transcriptional program [29]. The discovery that HDAC1‐3 and SIRT2 can remove lactyl marks further indicates that lactylation is dynamically regulated and is not simply an irreversible chemical footprint of lactate accumulation [26, 59].

Functionally, acetylation may report acetyl‐CoA‐dependent carbon availability and transcriptional activation, whereas lactylation may report glycolytic overflow, lactate accumulation, hypoxia, inflammation, or tissue‐stress states. Non‐covalent lactate sensing provides rapid responses to local lactate gradients through MCTs, HCAR1, NDRG3, MAVS, and AARS‐family proteins, while lactylation provides a more durable chromatin‐ or proteome‐level record of lactate‐rich exposure.

## Author Contributions

**Han Wang**: writing –original draft. **Si‐Yuan Yang**: writing –original draft. **Wei Xu**: writing –original draft, writing –review and editing.

## Conflicts of Interest

The authors declare no conflicts of interest.

## Data Availability

Data sharing not applicable to this article as no datasets were generated or analysed during the current study.
